# An oncogenic role of *lncRNA SNHG1* promotes *ATG7* expression and autophagy involving tumor progression and sunitinib resistance of Renal Cell Carcinoma

**DOI:** 10.1038/s41420-024-02021-3

**Published:** 2024-06-08

**Authors:** Pei Tian, Jinxing Wei, Jing Li, Junkai Ren, Chaohong He

**Affiliations:** 1 Department of Urology, The Affiliated Cancer Hospital of Zhengzhou University & Henan Cancer Hospital, Zhengzhou, 450008 Henan Province PR China; 2 Department of Urology, First Affiliated Hospital of Zhengzhou University, Zhengzhou, 450052 Henan Province PR China

**Keywords:** Molecular biology, Cell biology, Cancer

## Abstract

Renal cell carcinoma (RCC) is a malignant tumor with high incidence in adult kidney. Long non-coding RNAs (lncRNAs) have recently been recognized as important regulators in the development of RCC. However, whether lncRNA SNHG1 is associated with RCC progression remains to be elucidated. Here, the role of SNHG1 in RCC autophagy and sunitinib resistance was evaluated. Expression of SNHG1 in RCC tissues and cells was assessed using RT-qPCR. Western blot was utilized to measure the levels of autophagy-related molecules and ATG7. RNA pull-down and RIP assays were performed to confirm the molecular axis between SNHG1/PTBP1/ATG7. Cell proliferation, migration, invasion and apoptosis were analyzed by CCK-8, EdU, transwell and flow cytometry, respectively. The subcellular localization of SNHG1 was determined by an intracellular fractionation assay. The fluorescence intensity of GFP-LC3 autophagosome in RCC cells was detected. IHC staining was performed to test ATG7 expression in tumor tissues from nude mice. Here, a positive correlation of upregulated SNHG1 with poor prognosis of RCC patients was observed in RCC tissues and cells. SNHG1 knockdown suppressed tumor growth and reversed sunitinib resistance and autophagy of RCC cells. Additionally, SNHG1 was found to directly bind to PTBP1, thereby positively regulating ATG7 expression. Furthermore, we verified that SNHG1 mediated the malignant behavior of RCC cells through the PTBP1/ATG7 axis. To sum up, SNHG1 regulates RCC cell autophagy and sunitinib resistance through the PTBP1/ATG7 axis, which highlights a promising therapeutic target for RCC treatment.

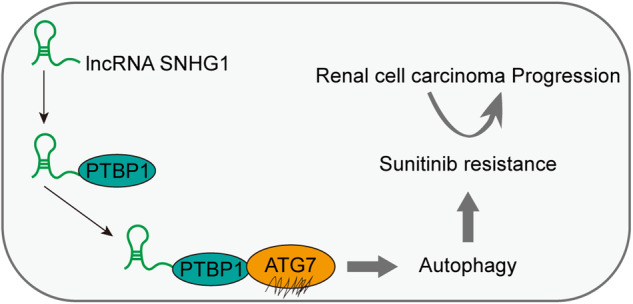

## Introduction

Renal cell carcinoma (RCC) is one of the adult renal malignancies originating from renal tubular epithelial cells [1]. RCC has become the most common renal malignancy, accounting for 85% of all renal malignancies [2]. The incidence of RCC is increasing worldwide. Currently, targeted drugs for RCC, such as sunitinib, have been used to treat patients with advanced RCC [3]. Sunitinib is a protein tyrosine kinase inhibitor with anti-tumor and anti-angiogenesis effects [4]. However, 30% of RCC patients develop primary resistance to sunitinib, and most patients become resistant to sunitinib within 6–15 months [5]. The overall survival rate of RCC patients remains a major problem currently facing. Therefore, it is necessary to elucidate the mechanism underlying RCC progression and sunitinib resistance.

Autophagy is an important process involved in regulating tumor resistance to multiple chemotherapeutic drugs [6, 7]. Autophagy plays a crucial role in RCC progression and sunitinib resistance. For example, ZHX2 enhances sunitinib resistance by regulating self-protective autophagy in RCC [8]. Combination therapy of sunitinib and chloroquine inhibits autophagy and induces apoptosis in RCC cells [9]. Nevertheless, the role of autophagy in RCC requires further investigation. The molecular mechanism of autophagy involves several conserved autophagy-related genes (ATGs), which have diverse functions in various physiological contexts [7]. Among these genes, ATG7 interacts with LC3b and is embedded in the early steps of autophagosome formation, which has been identified to play a role in a variety of physiological and pathological conditions [10]. It has been shown that ATG7 is essential for the tumorigenesis of RCC. For instance, ATG7 overexpression increases the level of LC3II in RCC cells [11]. Nevertheless, the precise mechanism underlying the involvement of ATG7 in RCC progression remains elusive.

Long non-coding RNAs (lncRNAs) are composed of more than 200 nucleotides and play important roles in autophagy regulation in a variety of cancers [12, 13]. Particularly, lncRNAs are involved in the progression of RCC [14, 15]. LncRNA SNHG1 is located on chromosome 11q12.3 and has been confirmed to be involved in multiple cellular processes, including autophagy [16]. More recently, Ye et al. showed that SNHG1 is upregulated in RCC cells and tissues, and SNHG1 promotes the malignant characteristics of RCC cells [17]. Moreover, exosomal SNHG1 extracted from cancer-associated fibroblasts enhances RCC progression [18]. Additionally, our previous study found that SNHG1 upregulated STAT3 via targeting miR-129-3p, thereby promoting PD-L1-mediated immune escape of RCC cells, indicating that SNHG1 exerts an oncogene biological function in RCC [19]. Nevertheless, the specific mechanism of SNHG1 in RCC remains unclear. Previous studies have shown that nearly 20–30% of human lncRNAs can bind to RNA-binding proteins (RBPs) to mediate the expression changes of downstream target genes in the “lncRNA-RBP” pattern [20]. Bioinformatic databases predicted a potential binding relationship between SNHG1 and PTBP1, a RBP with various molecular functions related to RNA metabolism [21]. Therefore, we speculated that SNHG1/PTBP1 might be involved in RCC progression.

Therefore, we explored the role of SNHG1 in regulating RCC. We propose that SNHG1 can promote the stability of ATG7 mRNA, regulate autophagy, and mediate RCC progression and sunitinib resistance by binding to PTBP1. Our present study may provide potential biomarkers for sunitinib resistance in RCC patients.

## Results

### High expression of SNHG1 indicated poor prognosis of RCC patients

**Fig. 1:**
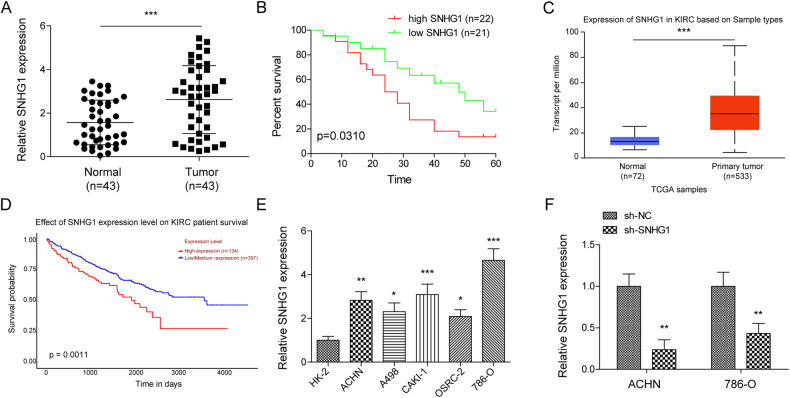
High expression of SNHG1 indicated poor prognosis of RCC patients. **A** The SNHG1 levels in 43 RCC tissues and matched tumor-adjacent normal tissues were analyzed using RT-qPCR. **B** Kaplan–Meier with log-rank test showed the overall survival in RCC patients according to the expression of SNHG1. **C** SNHG1 expression in RCC tissues was higher than that in normal tissues by analyzing TCGA. **D** The correlation between SNHG1 expression and the survival prognosis of RCC patients in TCGA database. **E** RT-qPCR was used to evaluate SNHG1 expression in normal HK-2 cells and RCC cell lines (ACHN, A498, CAKI-1, OSRC-2, 786-O). **F** ACHN and 786-O cells were transfected with sh-NC or sh-SNHG1. Transfection efficiency was verified by RT-qPCR. Bars represent mean ± SD. Experiments were repeated at least three times. *P < 0.05, **P < 0.01, ***P < 0.001.

We first examined the expression pattern of SNHG1 on the basis of obtaining tumor tissues from RCC patients. As illustrated in Fig. 1A, RT-qPCR analysis demonstrated that, compared to the matched adjacent normal tissues, SNHG1 levels in RCC tissues were remarkably increased. Moreover, survival analysis showed that patients with high SNHG1 expression displayed poor survival prognosis, suggesting that SNHG1 was positively correlated with poor prognosis of RCC patients (Fig. 1B). According to the TCGA database analysis, SNHG1 was highly expressed in RCC (Fig. 1C). Meanwhile, the correlation between SNHG1 level and survival prognosis of RCC patients in TCGA database was consistent with our above results (Fig. 1D). Furthermore, SNHG1 level in RCC cells was evaluated by RT-qPCR, and the data presented that SNHG1 expression was generally increased in RCC cell lines, compared with normal HK-2 cells (Fig. 1E). These findings preliminarily validate the potential oncogenic role of SNHG1 involved in RCC progression. On this basis, ACHN and 786-O cells with the most significant upregulation of SNHG1 were chosen for the following experiments. Subsequently, RCC cells were treated with shRNA specifically targeting SNHG1, and the transfection efficiency was verified by RT-qPCR. Transfection of sh-SNHG1 resulted in downregulation of SNHG1 in both ACHN and 786-O cells (Fig. 1F).

### SNHG1 knockdown inhibited RCC cell proliferation, migration and invasion

**Fig. 2:**
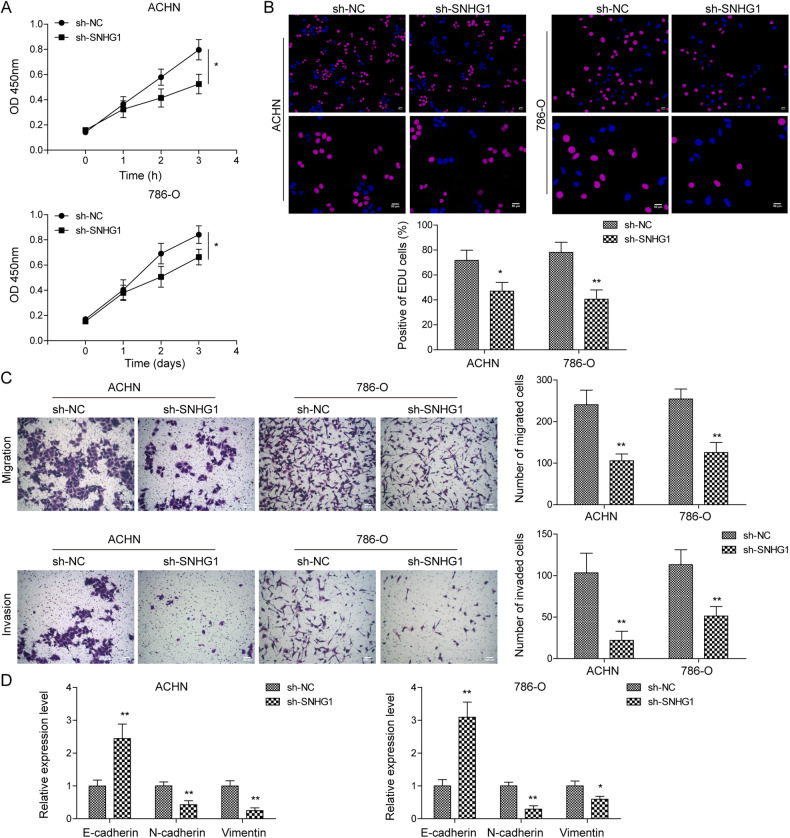
SNHG1 knockdown inhibited RCC cell proliferation, migration and invasion. ACHN and 786-O cells were transfected with sh-NC or sh-SNHG1 for 48 h. After that, CCK-8 and EdU assays were performed to determine cell viability (**A**) and proliferation (**B**). Scale bar, 50 μm. Transwell assay was utilized to assess cell migration and invasion (**C**). Scale bar, 100 μm. **D** RT-qPCR was used to detect EMT-related markers (E-cadherin, N-cadherin, Vimentin) in ACHN and 786-O cells with sh-NC or sh-SNHG1. Values are mean ± SD. Experiments were repeated at least three times. *P < 0.05, **P < 0.01.

Next, we explored the biological function of SNHG1 in RCC cell progression. In ACHN and 786-O cells stably transfected with sh-NC or sh-SNHG1, CCK-8, EdU, and transwell assays were performed for assessing the capacities of cell proliferation, migration and invasion. As shown in Fig. 2A–C, knockdown of SNHG1 significantly inhibited RCC cell proliferation, migration and invasion. Meanwhile, compared to sh-NC group, SNHG1 silencing obviously suppressed the protein expression of N-cadherin and Vimentin, but increased the expression of E-cadherin in ACHN and 786-O cells (Fig. 2D). To sum up, the above data suggest that SNHG1 is involved in RCC growth and invasion.

### SNHG1 knockdown impaired sunitinib resistance and autophagy in RCC cells

**Fig. 3:**
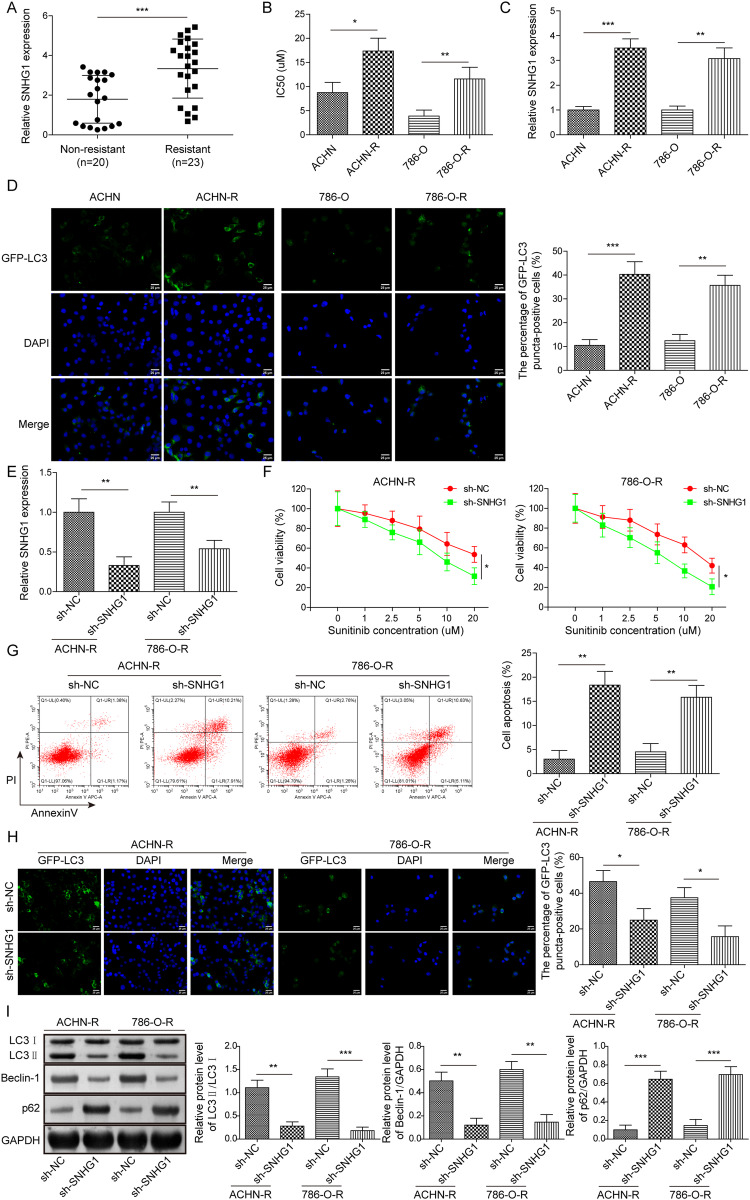
SNHG1 knockdown impaired sunitinib resistance and autophagy in RCC cells. **A** RT-qPCR was used to examine SNHG1 expression in non-resistant RCC tissues (*n* = 20) and resistant RCC tissues (*n* = 23). **B** MTT assay was conducted to test the IC_50_ of sunitinib-treated RCC cells, as well as their corresponding resistant cells transfected with sh-SNHG1 or sh-NC. **C** RT-qPCR was performed to determine SNHG1 expression in RCC cells and RCC cell resistant strains. **D** RCC cells and RCC cell resistant strains were transfected with GFP-LC3 for 48 h. The fluorescence intensity of GFP-LC3 autophagosomes was detected. Scale bar, 25 μm. **E** Transfection efficiency of SNHG1 silencing vector in RCC cell resistant strains was detected by RT-qPCR. **F** MTT assay was conducted to assess cell viability in RCC cell resistant strains with sh-NC or sh-SNHG1. **G** Flow cytometric detection of cell apoptosis in RCC cell resistant strains with sh-NC or sh-SNHG1. **H** RCC cell resistant strains were transfected with GFP-LC3 for 48 h. The fluorescence intensity of GFP-LC3 autophagosomes in cells with sh-NC or sh-SNHG1 was detected. Scale bar, 25 μm. **I** The protein levels of autophagy-related molecules were assessed by western blot in RCC cell resistant strains with sh-NC or sh-SNHG1. Error bars represent SD. Experiments were repeated at least three times. *P < 0.05, **P < 0.01, ***P < 0.001.

We further distinguished the differential expression of SNHG1 between the drug-resistant group and non-resistant group in clinical tissue samples. RT-qPCR analysis demonstrated that SNHG1 level was remarkably increased in sunitinib resistant RCC tissues compared to non-sunitinib resistant RCC tissues (Fig. 3A). Moreover, the respective drug-resistant strains of ACHN and 786-O cells were constructed to investigate the contribution of SNHG1 to sunitinib resistance in RCC. The IC_50_ values of RCC cell resistant strains treated with sunitinib were extremely higher than those of the respective drug-sensitive strains treated with sunitinib, suggesting sunitinib resistance (Fig. 3B). Compared with RCC cells, SNHG1 level was remarkably up-regulated in RCC cell resistant strains (Fig. 3C). Notably, compared with the drug-sensitive cell strains, the fluorescence intensity of GFP-LC3 autophagosomes was markedly enhanced in RCC cell resistant strains (Fig. 3D). Next, RCC cell resistant strains were transfected with sh-SNHG1. Decreased SNHG1 expression was observed in drug-resistant strains with sh-SNHG1 (Fig. 3E). Compared with sh-NC group, after knocking down SNHG1, the cell viability of RCC cell resistant strains was significantly repressed and the IC_50_ value of drug treatment was also greatly reduced (Fig. 3F). Moreover, the apoptosis rate of RCC cell resistant strains was increased greatly after SNHG1 silencing, compared with the control group (Fig. 3G). Furthermore, compared with sh-NC group, the fluorescence intensity of GFP-LC3 in RCC cell resistant strains was obviously weakened after knocking down SNHG1 (Fig. 3H). Western blot analysis further showed that the ratio of LC3II/LC3I and the level of Beclin1 were significantly decreased in cells with sh-SNHG1 compared to control cells, while the expression of p62 was upregulated (Fig. 3I). Importantly, the sunitinib resistance of RCC cells was significantly inhibited by SNHG1 silencing and treatment with autophagy inhibitor 3-methyladenine (3-MA) (Supplementary Fig. 1). Taken together, these findings reveal that SNHG1 mediates sunitinib resistance by affecting the autophagy activity of RCC cells.

### SNHG1 positively regulated ATG7 expression

**Fig. 4:**
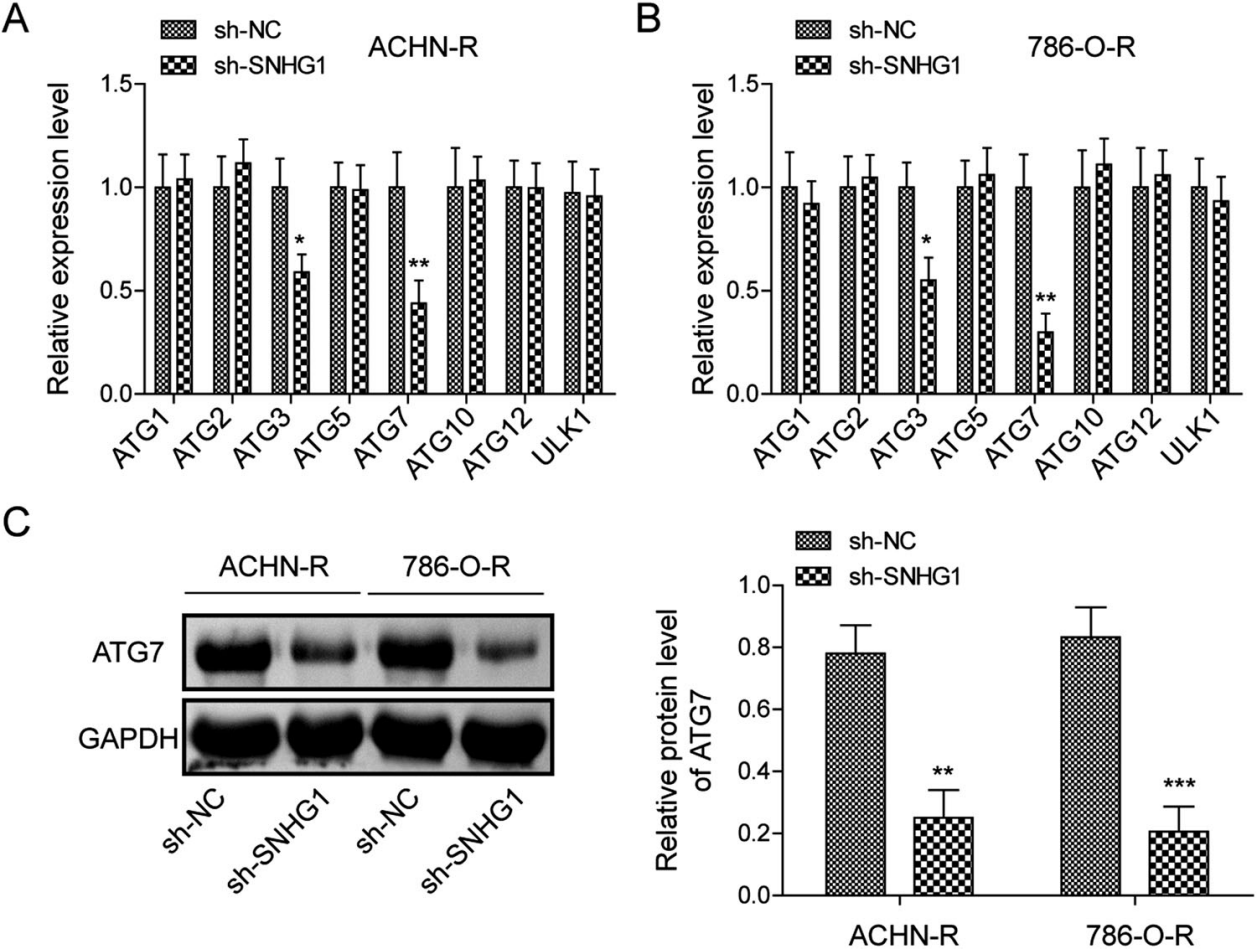
SNHG1 positively regulated ATG7 expression. **A**, **B** RT-qPCR was used to analyze the expression of autophagy-related molecules (ATG1\2\3\5\7\10\12 and ULK1) in ACHN-R and 786-O-R cells with sh-NC or sh-SNHG1. **C** Western blot was conducted to examine the ATG7 expression in ACHN-R and 786-O-R cells with sh-NC or sh-SNHG1. Values were expressed as mean ± SD. Experiments were repeated at least three times. *P < 0.05, **P < 0.01, ***P < 0.001.

Next, we explored the regulation of SNHG1 on the expression levels of autophagy-related molecules in RCC cell resistant strains. RT-qPCR results showed that among the autophagy-related molecules we screened, ATG7 and ATG3 levels were dramatically decreased in SNHG1 silenced cells (Fig. 4A, B). Therefore, the ATG7 with the greatest change, was selected for subsequent experiments. Moreover, Western blot was used to assess ATG7 protein levels after knocking down SNHG1. The results were consistent with the RT-qPCR results (Fig. 4C). Thus, these results suggest that SNHG1 exerts a positive regulation on ATG7 expression.

### ATG7 was essential for SNHG1-mediated malignant behaviors of RCC cells

**Fig. 5:**
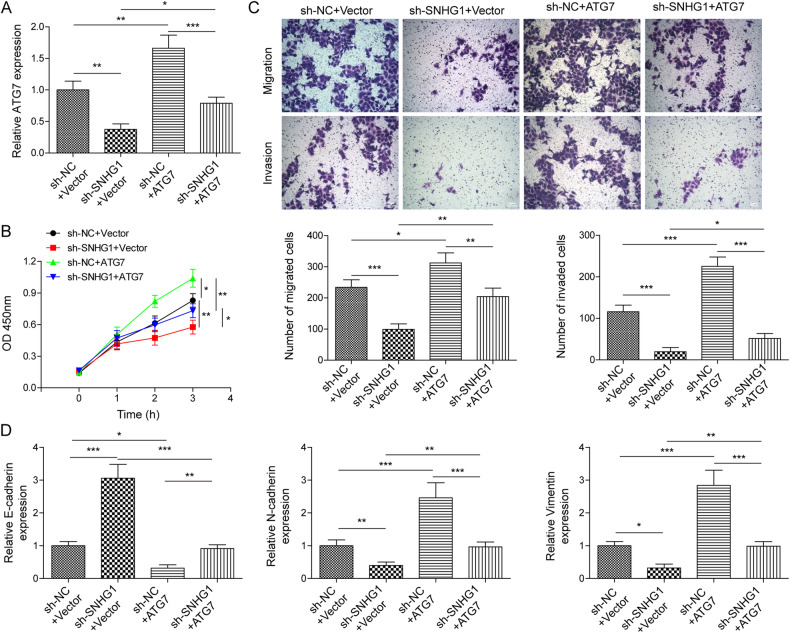
ATG7 was essential for SNHG1-mediated malignant behaviors of RCC cells. RCC cell resistant strains were transfected with sh-SNHG1, pcDNA3.1-ATG7 and negative controls. After 48 h transfection, (**A**) RT-qPCR was used to test ATG7 expression in RCC cell resistant strains with sh-SNHG1 and pcDNA3.1-ATG7. **B** CCK-8 analysis was performed to determine cell viability in different groups. **C** Transwell assay was utilized to assess cell migration and invasion in different groups. Scale bar, 100 μm. **D** RT-qPCR detection of EMT-related markers in different groups. Results expressed as mean ± SD. Experiments were repeated at least three times. **P < 0.01, ***P < 0.001.

To further clarify that ATG7 is a key molecule by which SNHG1 exerts its biological functions in RCC, ACHN cells transfected with sh-SNHG1 or sh-NC were exposed to ATG7 overexpression plasmids. When SNHG1 expression was down-regulated, ATG7 level was significantly decreased, which was rescued by co-transfection with overexpressed ATG7 (Fig. 5A). Compared to the control group, RCC cell proliferation was remarkably inhibited by SNHG1 depletion, ATG7 overexpression promoted a significant increase in cell proliferation activity, and overexpressed ATG7 also overturned the inhibitory effect of cell proliferation mediated by SNHG1 depletion (Fig. 5B). Additionally, RCC cell migration and invasion were markedly reduced in SNHG1-silenced cells; whereas RCC cell migration and invasion abilities were enhanced in ATG7-upregulated cells. However, overexpression of ATG7 overturned the inhibitory effect mediated by SNHG1 downregulation (Fig. 5C). Besides, SNHG1 depletion suppressed EMT in RCC cells, as confirmed by decreased N-cadherin and Vimentin levels, and increased E-cadherin expression. These results were reversed upon overexpression of ATG7 (Fig. 5D). Altogether, these findings reveal that ATG7 is essential for SNHG1-mediated malignant behaviors of RCC cells.

### ATG7 was essential for SNHG1-mediated sunitinib resistance and autophagy in RCC cells

**Fig. 6:**
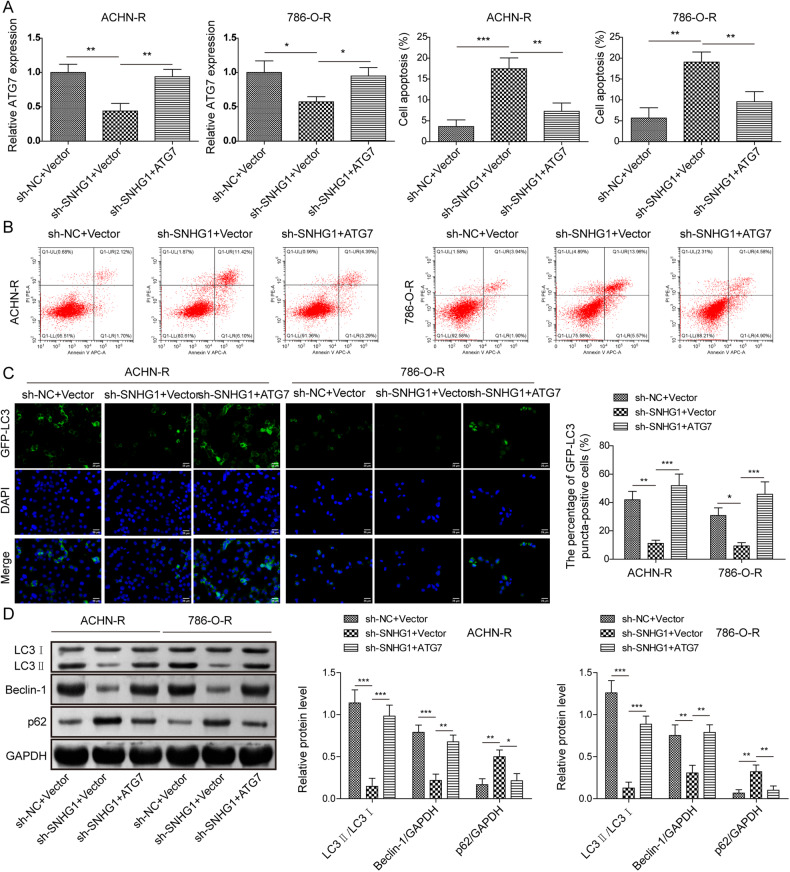
ATG7 was essential for SNHG1-mediated sunitinib resistance and autophagy in RCC cells. RCC cell resistant strains were transfected with sh-SNHG1, pcDNA3.1-ATG7 and negative controls. **A** RT-qPCR was used to test ATG7 expression in RCC cell resistant strains with sh-SNHG1 and pcDNA3.1-ATG7. **B** Flow cytometric detection of cell apoptosis in different groups. **C** The fluorescence intensity of GFP-LC3 autophagosomes was detected in different groups. Scale bar, 25 μm. **D** The protein levels of autophagy-related molecules were assessed by western blot in different groups. All data are represented as the mean ± SD. Experiments were repeated at least three times. *P < 0.05, **P < 0.01, ***P < 0.001.

We further verified whether ATG7 was involved in SNHG1-mediated autophagy and sunitinib resistance in RCC cells. ATG7 expression was suppressed by SNHG1 silencing, and it was recovered by ATG7 overexpression in sunitinib resistant RCC cells (Fig. 6A). As illustrated in Fig. 6B, compared to the control group, SNHG1 silencing was able to inhibit the apoptosis rate of RCC cell resistant strains, but this effect could be partially abrogated by reinforced ATG7. Meanwhile, compared with sh-NC group, the fluorescence intensity of GPF-LC3 was obviously weakened in RCC cell resistant strains with sh-SNHG1, and co-transfection of pcDNA3.1-ATG7 enhanced the fluorescence intensity of GFP-LC3 (Fig. 6C). Additionally, the ratio of LC3II/LC3I and the level of Beclin1 in SNHG1 knockdown cells were greatly decreased, while p62 level was increased. On the other hand, ATG7 overexpression remarkably abrogated these effects (Fig. 6D). The above results indicate that ATG7 is essential for SNHG1-mediated sunitinib resistance and autophagy in RCC cells.

### SNHG1 exerted an oncogenic role by increasing ATG7 expression through binding to PTBP1

**Fig. 7:**
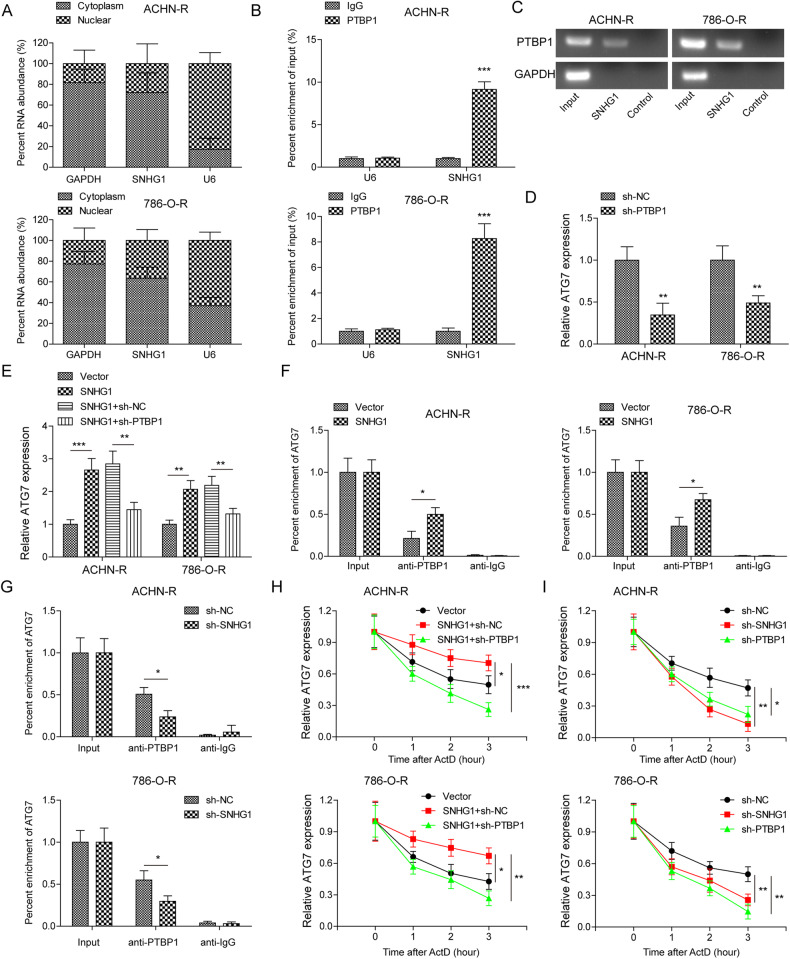
SNHG1 exerted an oncogenic role by increasing ATG7 expression through binding to PTBP1. **A** Subcellular fractionation assay was used to detect the expression and location of SNHG1 in RCC cell resistant strains. **B**, **C** RNA pull-down and RIP assays verified the binding relationship between SNHG1 and PTBP1 in RCC cell resistant strains. **D** RT-qPCR was performed to detect ATG7 expression in RCC cell resistant strains with sh-NC or sh-SNHG1. **E** RT-qPCR was conducted to measure ATG7 expression in RCC cell resistant strains with pcDNA3.1-SNHG1, sh-PTBP1 and negative controls. **F**, **G** RIP was utilized to test the effect of SNHG1 on the recruitment of PTBP1 to ATG7 mRNA in RCC cell resistant strains. **H**, **I** RT-qPCR analysis of the half-life of ATG7 mRNA in RCC cell resistant strains with pcDNA3.1-SNHG1, sh-PTBP1 and negative controls. All data are represented as mean ± SD. Experiments were repeated at least three times. *P < 0.05, **P < 0.01, ***P < 0.001.

We further explored the molecular mechanism of ATG7 regulation by SNHG1 in RCC, and found that SNHG1 was distributed in both cytoplasm and nucleus, and mainly in the cytoplasm of RCC cell resistant strains (Fig. 7A). Next, RNA pull-down and RIP assays verified the binding of SNHG1 to PTBP1 in RCC cell resistant strains (Fig. 7B, C). Notably, after silencing PTBP1, the expression of ATG7 was significantly decreased (Fig. 7D). Moreover, after overexpressing SNHG1, the expression of ATG7 was obviously increased compared to the control. However, when PTBP1 was downregulated, the expression of ATG7 was suppressed (Fig. 7E). Additionally, the relative enrichment of ATG7 with PTBP1 was reduced after knockdown of SNHG1 (Fig. 7F, G). Next, we treated sunitinib resistant cells with actinomycin D (ActD), which is widely used in experiments to block the transcription process [22]. We found that silencing of SNHG1 or PTBP1 increased the degradation of ATG7 mRNA. Overexpression of SNHG1 delayed the half-life of ATG7 mRNA. When silencing PTBP1 at the same time, the half-life of ATG7 mRNA returned to close to normal level (Fig. 7H, I). The above data suggest that SNHG1 exerts its oncogenic role via increasing ATG7 expression by binding to PTBP1.

### SNHG1 knockdown suppressed tumor growth and reversed sunitinib resistance and autophagy

**Fig. 8:**
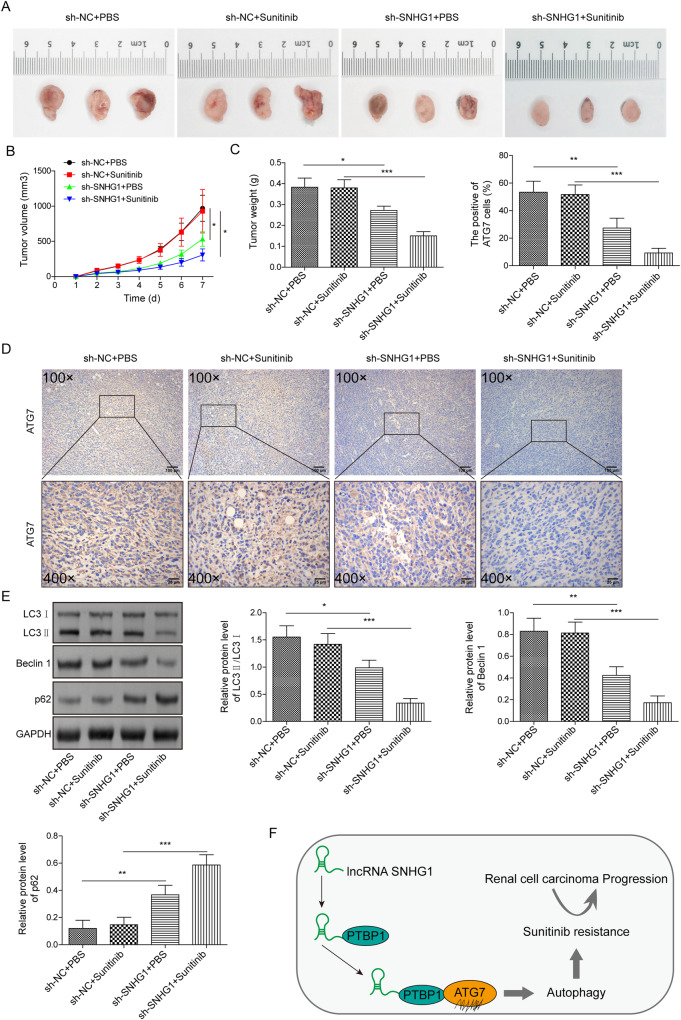
SNHG1 knockdown suppressed tumor growth and reversed sunitinib resistance and autophagy. Nude mice were injected subcutaneously with the stable ACHN-R cells transfected with vector or sh-SNHG12 (*n* = 8/group). From day 20, sunitinib was fed to mice every day. **A** Images of the xenograft tumors in different groups. **B** The tumor volume was measured in different groups. **C** The tumor weight was detected in different groups. **D** IHC analysis was conducted to test ATG7 expression in tumor tissues of nude mice. Scale bar, 25 μm. **E** The protein levels of autophagy-related molecules were assessed by western blot in tumor tissues of different groups. **F** Molecular mechanism diagram of SNHG1 in modulating RCC progression. Error bars stand for the mean ± SD of at least triplicate experiments. *P < 0.05, **P < 0.01.

To explore the role of SNHG1 in RCC progression, we injected into the nude mice subcutaneously with the stable ACHN-R cells transfected with vector or sh-SNHG12. From day twenty, sunitinib was fed to mice every day. Meanwhile, SNHG1 knockdown suppressed the growth of RCC xenografts (Fig. 8A–C). Furthermore, IHC analysis revealed that ATG7 expression was remarkably decreased in tumor tissues with SNHG1 silencing (Fig. 8D). Western blot analysis further demonstrated that the LC3II/LC3I ratio and Beclin1 were downregulated and p62 was upregulated in tumor tissues with SNHG1 knockdown, and sunitinib treatment enhanced the effect of sh-SNHG1 (Fig. 8E). Therefore, these results demonstrated that SNHG1 knockdown suppressed tumor growth and reversed sunitinib resistance and autophagy. As shown in Fig. 8F, we generated a schematic diagram of this regulatory process, describing that SNHG1 induced ATG7 expression via binding to PTBP1, thereby promoting RCC progression and sunitinib resistance.

## Discussion

RCC is a highly aggressive cancer with unremarkable initial symptoms [23]. Nearly 30% of RCC patients have developed metastasis at the time of first diagnosis [24]. Although targeted drugs, such as sunitinib, have been used to treat patients with advanced RCC, unfortunately, tumor resistance usually occurs within 6–15 months and the overall survival of patients remains a major problem currently facing [25, 26]. The present study elucidated the contribution of lncRNA SNHG1 to RCC. We identified that SNHG1 could regulate RCC cell autophagy and sunitinib resistance through the PTBP1/ATG7 axis, providing a new therapeutic opportunity for RCC chemoresistance.

Autophagy is a highly conserved physiological phenomenon that breaks down unnecessary or dysfunctional components [27]. Accumulating evidence suggests that autophagy has an oncogenic role in RCC [28]. For instance, TRPM3 and miR-204 regulate oncogenic autophagy in RCC [29]. In addition, autophagy is a life activity by which tumors develop resistance to a variety of chemotherapeutic drugs [30, 31]. For example, ASPP2 silencing leads to increased gemcitabine resistance, which is attributed to enhanced autophagy in pancreatic cancer [32]. Besides, miR-541 enhances the response of hepatocellular carcinoma to sorafenib treatment by inhibiting autophagy [33]. Moreover, enhanced autophagy contributes to increased drug resistance in breast cancer [34]. Consistently with previous findings, our data indicated that sunitinib-resistant RCC cells had increased autophagic activity, and further confirmed SNHG1 as an important autophagy regulatory lncRNA that enhanced RCC resistance to sunitinib by inducing autophagy.

LncRNAs are essential for human cancer progression through the lncRNA-RBP network and competing endogenous RNA (ceRNA) network [20, 35]. For instance, lncRNA HOTAIR enhances sunitinib resistance via targeting miR-17-5p in RCC cells [36]. LncRNA URRCC promotes RCC cell proliferation and metastasis [37]. SNHG1 is a novel oncogenic lncRNA aberrantly expressed in different cancers including colorectal, liver and esophageal cancers [38]. Supporting our findings, a previous research demonstrated that SNHG1 is significantly upregulated in RCC, and highly expressed SNHG1 predicts poor prognosis [39]. We previously confirmed that SNHG1 is involved in regulating RCC in a classical ceRNA regulatory pattern [19]. The present study is the first to evaluate the correlation between SNHG1 and RBP in RCC. PTBP1 is a RBP involved in tumorigenesis, including RCC. Shan et al. reported that PTBP1 depletion reduces RCC cell proliferation and angiogenesis [40]. Besides, SNHG1 regulates rheumatoid synovial invasion and proliferation by interacting with PTBP1 [41]. Moreover, SNHG1 regulates adipogenic differentiation of bone marrow-derived mesenchymal stem cells through interacting with PTBP1 [42]. In our study, we discovered that SNHG1 played a fundamental role in sunitinib resistance of RCC by targeting PTBP1.

ATG7 is involved in the early stages of autophagosome formation [43], and ATG7 is important for homeostasis maintenance and disease development [44]. In addition, ATG7 is found to be frequently associated with RCC, and its low expression is correlated with RCC progression [45]. Meanwhile, inhibition of autophagy significantly improves the sensitivity of breast cancer to sunitinib and erlotinib [46]. Here, we revealed that SNHG1 bound to PTBP1, and delayed the degradation of ATG7 mRNA, indicating the important value of the SNHG1-PTBP1-ATG7 axis in RCC autophagy and sunitinib resistance.

Collectively, we demonstrated the role of SNHG1 as a tumor inducer in RCC chemoresistance. SNHG1 silencing reversed the resistance to sunitinib in sunitinib resistant RCC cells by reducing autophagy. Mechanically, high expression of SNHG1 in RCC cells recruited PTBP1. PTBP1 bound to ATG7 and activated its expression, thereby promoting autophagy and sunitinib resistance. These findings may provide a novel perspective to uncover the molecular mechanisms of sunitinib resistance and provide new insights into RCC treatment. Nevertheless, there are still some limitations in our study. For example, Beclin1 helps in the nucleation and formation of phagophore and ATG7 works in the elongation of phagophore. Due to the limited conditions of our laboratory, the ultra structure of phagophore cannot be provided at present. Further research should be performed to explore how Beclin1 expression is downregulated in sh-SNHG1 cells if SNHG1 directly regulates autophagy by modulating ATG7. In addition, in the future, our research should also be confirmed by using an autophagy activator, such as Rapamycin, to further verify that SNHG1 directly affects autophagy through ATG7.

## Materials and methods

### Clinical specimens

RCC tissues and adjacent non-cancerous renal tissues were acquired from 43 cases aged 36–72 (mean, 57) years undergoing surgical resection for RCC at The Affiliated Cancer Hospital of Zhengzhou University & Henan Cancer Hospital. Informed consent was obtained from all patients. The fresh tissues were stored in liquid nitrogen. The study was approved by the Clinical Research Ethics Committee of The Affiliated Cancer Hospital of Zhengzhou University & Henan Cancer Hospital. None of the patients received pre-operative treatment. The clinical information of 43 clinical cases was listed in Supplementary Table 1.

### Cell culture

The human renal tubular epithelial cells (HK-2) as well as five human RCC cell lines (A498, ACHN, OSRC-2, 786-O, CAKI-1) were obtained from American Type Culture Collection (ATCC, Manassas, VA, USA). All cells had been authenticated before use, and then were cultured in DMEM medium (Invitrogen, Carlsbad, CA, USA) supplemented with 10% FBS (Invitrogen) and 1% penicillin/streptomycin (Sigma, St. Louis, MO, USA) in humidified air at 37 °C with 5% CO_2_. Cells were treated with 2 μg/mL actinomycin D (ActD) (Sigma) as a transcription inhibitor.

### Cell transfection

Short hairpin RNAs specifically targeting SNHG1 (termed sh-SNHG1) and PTBP1 (termed sh-PTBP1) were obtained from Guangzhou RiboBio (RiboBio, Guangzhou, China), and the sequences were listed in Supplementary Table 2. Additionally, the pcDNA3.1 vector (GenePharma, Shanghai, China) containing the full-length cDNA sequence of SNHG1 or ATG7 was used to overexpress SNHG1 or ATG7. Transfection was performed using Lipofectamine 2000 reagent (Invitrogen) according to the manufacturer’s instruction for 48 h.

### Construction of resistant strains and MTT detection of IC_50_

The sunitinib-resistant cells (ACHN-R, 786-O-R) were established as previously described [47]. For MTT assay, cells were seeded into 96-well plates (5 × 10^3^ cells/well) for 48 h. 100 μl MTT (5 mg/mL, Sigma) was added to each well and incubated for additional 4 h at 37 °C, followed by staining with 100 μl of DMSO. The absorbance at 570 nm was recorded with a microplate reader (Molecular Devices, Sunnyvale, CA, USA). The half‑maximal inhibitory concentration (IC_50_) value was calculated.

### CCK-8 and EdU assays

To detect cell viability, the transfected cells were seeded into 96-well plates at a density of 5000 cells/well. 10 μl CCK-8 reagent (Beyotime, Shanghai, China) was added to each well after incubation for 24, 48, 72 and 96 h, and cells were incubated for 2 h at 37 °C. The optical density value was read by a microplate reader (Molecular Devices) at 450 nm. This work utilized 5-Ethynyl-2′-deoxyuridine (EdU) Kit (RiboBio) for assessing cell proliferation. In brief, EdU was used to incubate cells for a 2-h period, followed by DAPI staining (Invitrogen). Finally, fluorescence microscopy (Olympus, Tokyo, Japan) was utilized to observe cells.

### Transwell assay

200 μl of cell solution (1 × 10^5^/mL) was added into the upper chamber of Transwell chambers (Corning, NY, USA) with serum-free medium. Culture medium containing 10% FBS was added into the lower chamber. After incubation for 36 h, the migrated cells were fixed with 4% paraformaldehyde for 15 min and stained with 0.1% crystal violet for 5 min. For invasion analysis, transwell chambers were precoated with Matrigel (BD Biosciences, San Jose, CA, USA), and the remaining steps were syngeneic to cell migration experiments. Finally, the number of migrated or invaded cells from five random fields was counted under a light microscope (Olympus).

### Immunofluorescence detection of LC3

Cells were transfected with 1 μg of GFP-LC3 (GenePharma, Shanghai, China), with 2 μl of Lipofectamine 2000 (Invitrogen) in 100 μl of OptiMEM and incubated for 48 h. Cells were then fixed in 4% formaldehyde solution for 30 min. After staining with DAPI for 5 min, cells were observed under fluorescence microscope (Olympus).

### Flow cytometry

1 × 10^6^ cells were washed twice using PBS and resuspended in 1X binding buffer (BD Biosciences, Franklin Lakes, NJ, USA). Apoptotic cells were measured by a flow cytometer (BD Biosciences) after staining with Annexin V-FITC and propidium iodide (PI) (Thermo Fisher Scientific, Waltham, MA, USA) according to the manufacturer’s protocol.

### Real-time quantitative PCR (RT-qPCR)

Total RNA was extracted from tissues or cells using Trizol reagent (Sigma). The cDNA was synthesized with PrimeScript RT reagent Kit (Takara, Kyoto, Japan). Real-time PCR was performed using the SYBR Green PCR Kit (Takara). The targeted gene expressions were normalized to that of the internal control gene, GAPDH. The correlated primer sequences were displayed in Supplementary Table 3. Relative changes in gene expression were computed using the 2^−∆∆Ct^ formula.

### Western blot analysis

Cells were lysed in ice cold RIPA lysis buffer (Sigma). Protein concentrations were measured using the Bradford assay (Beyotime). Equal proteins were run on 10% SDS-PAGE gels and were electro-transferred to PVDF membranes (Millipore, Boston, MA, USA). Membranes were blocked with 5% skim milk for 1 h and then blocked with first antibodies at 4 °C overnight: Beclin1 (#3738, 1:1000, Cell Signaling Technology, CST, Danvers, MA, USA), LC3II/LC3I (#4108, 1:1000, CST), p62 (ab91526, 1:1,000, Abcam), ATG7 (#2631, 1:1000, CST), GAPDH (ab9485, 1:5000, Abcam). After rinsing with TBST, membranes were subject to 1-h incubation using HRP-labeled secondary antibodies (#7074, 1:1000, CST) at room temperature. Finally, ECL detection kit (Millipore) was utilized to visualize target signals. Bands were analyzed with ImageJ software (NIH, USA).

### Subcellular fractionation assay

Cell fractionation was carried out using the PARIS™ Kit (Thermo Fisher Scientific, USA) in 1 × 10^4^ cells. Firstly, cell fractionation buffer was used to resuspend the collected cells. Then, cells were placed on ice for 10 min. After centrifugation, the cell disruption buffer was utilized to conserve the nuclear pellet and supernatant for the extraction of RNA. SNHG1 contents were measured in the indicated fractions by real-time PCR. The GAPDH and U6 transcripts were used as internal markers to evaluate the fractioning efficiency.

### RNA pull-down

Biotin-labeled PTBP1 was transfected into cells using Lipofectamine 2000 reagent (Invitrogen). Cells were then lysed and RNA-protein complexes were incubated with streptavidin magnetic beads (Invitrogen) for 2 h. The precipitated components were purified with biotin elution buffer. The pulled down SNHG1 RNA was measured by RT-qPCR.

### RNA immunoprecipitation (RIP) assay

Magna RIP RNA-binding protein immunoprecipitation kit (Millipore) was used to confirm the combination of SNHG1 and PTBP1. After harvesting the cells in RIP lysis buffer, cell lysates were collected and added to magnetic beads coupled to anti-PTBP1 (Abcam) or immunoglobulin G (IgG) (Millipore) overnight at 4 °C. Later, SNHG1 was analyzed using RT-qPCR.

### Nude mice xenograft

The 4-week-old BALB/c male nude mice were obtained from Shanghai Laboratory Animal Center of Chinese Academy of Sciences (Shanghai, China) (*N* = 8/group) [48]. sh-SNHG1 or sh-NC was transfected into ACHN-R cells. Each nude mouse was later subcutaneously implanted with 3 × 10^6^ cells, within the principle of random allocation. On the 20th day, the animals were fed with vehicle or sunitinib. The tumor volume was detected at 2-/3-day intervals beginning at tumor occurrence to the 40th day. The investigator was blinded to the group allocation during the experiment. After 40 days, tumors were dissected and the weights were measured. Tumor volume = (Width^2^ × Length)/2. Each animal procedure gained approval from Animal Experimental Ethics Committee of The Affiliated Cancer Hospital of Zhengzhou University & Henan Cancer Hospital.

### Immunohistochemistry (IHC)

Tissue slides were deparaffinized and rehydrated. Endogenous peroxidase was blocked with 3% hydrogen peroxide for 30 min. Slides were blocked in 10% BSA for 10 min, and incubated with primary antibodies against ATG7 (1:200, Invitrogen) at 4 °C overnight, followed by incubating with the corresponding secondary antibody. Then, the sections were washed thoroughly and stained with diaminobenzidine (Sigma). Finally, images were observed using Olympus microscope.

### Statistical analysis

The clinical information of RCC patients were collected from The Cancer Genome Atlas (TCGA) database (http://cancergenome.nih.gov/). Statistical analyses were processed using GraphPad Prism 7 (Graphpad, La Jolla, CA, USA). Data are shown as mean ± standard deviation (SD) from at least three independent experiments. All data was in a normal distribution, and variance was similar between the groups that are being statistically compared. Student’s *t*-test was performed to compare the difference between two groups, and One-way ANOVA was utilized for multiple-group comparison. Kaplan-Meier with log-rank test was performed for survival analysis. *P* < 0.05 was considered statistically significant.

### Supplementary information

Supplementary tables and figures

Supplementary materials for western blot

## Data Availability

All data are available within the article and supplementary files, or from the authors upon reasonable request.
