# Supplementary material for: An oncogenic role of lncRNA SNHG1 promotes ATG7 expression and autophagy involving tumor progression and sunitinib resistance of Renal Cell Carcinoma
Source: Cell Death Discov. 2024 Jun 8;10:273. doi: 10.1038/s41420-024-02021-3 (PMC11162435; doi:10.1038/s41420-024-02021-3)
Supplement: Supplementary file 1 — Supplementary tables and figures [file 41420_2024_2021_MOESM1_ESM.pdf]

Supplementary figure legends

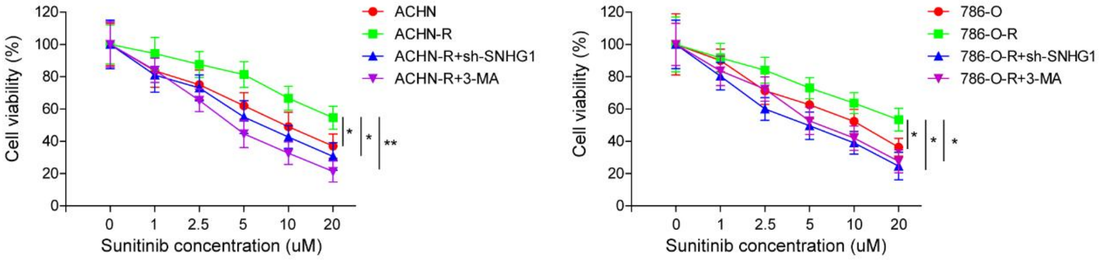

Supplementary figure 1. SNHG1 suppression and autophagy inhibitor contributed to sunitinib resistance in RCC. RCC resistant strains were transfected with sh-SNHG1 or treated with 3-methyladenine (3-MA, autophagy inhibitor, #S2767, Selleck Chemicals, Houston, TX) for 24 h. Cell viability was assayed by MTT assay. Error bars stand for the mean ± SD of at least triplicate experiments. *$P<0.05$, **$P<0.01$.

Supplementary table 1. Correlation between SNHG1 expression and clinicopathological parameters RCC patients

Parameter	Number	SNHG1 mRNA expression		P value
		Low (n=21)	High (n=22)	
Age (years)				0.7546
<57	16	7	9	
≥57	27	14	13	
Gender				0.0666
Female	18	12	6	
Male	25	9	16	
T stage				0.0268
T1 or T2	28	10	18	
T3 or T4	15	11	4	
N stage				0.0002
N0 or NX	25	6	19	
N1	18	15	3	
M stage				0.0022
M0 or MX	23	6	17	
M1	20	15	5	
G stage				0.0329
G1 or G2 or GX	24	8	16	
G3 or G4	19	13	6	
TNM stage				0.0005
I+II	26	7	19	
III+IV	17	14	3	

Supplementary table 2. The sequences of shRNAs used in this study.

**shRNAs**	**Sequence (5' to 3')**
sh-SNHG1	GGCCACGACCTTCTCTAAACTCGAGTTTAGAGAGAAGGTGCTGGCCTTTTT
	TGCTGTTGACAGTGAGCGCAGGATTCAAGTTCTTCCAGAATAGTGAAGCCACAGATGTA
sh-PTBP1	TTCTGGAAGAACTTGA ATCCTTTGCCTACTGCCTCGGA

Supplementary table 3. The primer sequences applied for RT-qPCR

SNHG1	F: 5’-ACGTTGGAACCGAAGAGAGC-3’	R: 5’-GCAGCTGAATTCCCCAGGAT-3’
ATG7	F: 5’-CCAGTGACGCCAGATTTCC-3’	R: 5’-GGCAGGCACAGATGCTATG-3’
ATG1	F: 5’-CAAGGTGGTGTTCTCTGTGC-3’	R: 5’-ACACATTCATGCAACCCACC-3’
ATG2	F: 5’-TGTATCCAGATGGGGGTGTT-3’	R: 5’-GGAACTTAAGCTGCCCTTGA-3’
ATG3	F: 5’-TGTTTGGCTATGATGAGCAACG-3’	R: 5’-CACATGGGAGGTGGTGGC-3’
ATG5	F: 5’-GATCACAAGCAACTCTGGATGG-3’	R: 5’-AGCCACAGGACGAAACAGC-3’
ATG10	F: 5’-AGACCATCAAAGGACTGTTCTGA-3’	R: 5’-GGGTAGATGCTCCTAGATGTGAC-3’
ATG12	F: 5’-AGAGCGAACACGAACCATCC-3’	R: 5’-CCCACGCCTGAGACTTGC-3’
ULK1	F: 5’-CAAGAAGAACCTCGCCAAGTC-	R: 5’-GGAAGAGCCTGATGGTGTCC-3’

	3’	
E-cadherin	F: 5’-CTGTGCCCAGCCTCCATGTTTT-3’	R: 5’-CTGGATAGCTGCCCATTGCAAGTTA-3’
N-cadherin	F: 5’-GTGCATGAAGGACAGCCTCT-3’	R: 5’-CCACCTTAAAATCTGCAGGC-3’
Vimentin	F: 5’-CCGGTGCAATCGTGATCTCTGGG-3’	R: 5’-ATTCAAGTCTCAGCGGGCTC-3’
GAPDH (an internal control)	F: 5’-GAAGGTGAAGGTCGGAGTC-3’	R: 5’-GAAGATGGTGATGGGATTTC-3’
