# Supplementary figures and images for: An oncogenic role of lncRNA SNHG1 promotes ATG7 expression and autophagy involving tumor progression and sunitinib resistance of Renal Cell Carcinoma
Source: Cell Death Discov. 2024 Jun 8;10:273. doi: 10.1038/s41420-024-02021-3 (PMC11162435; doi:10.1038/s41420-024-02021-3)

FIG3I

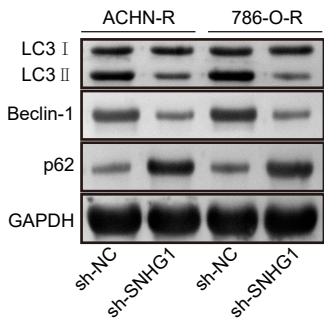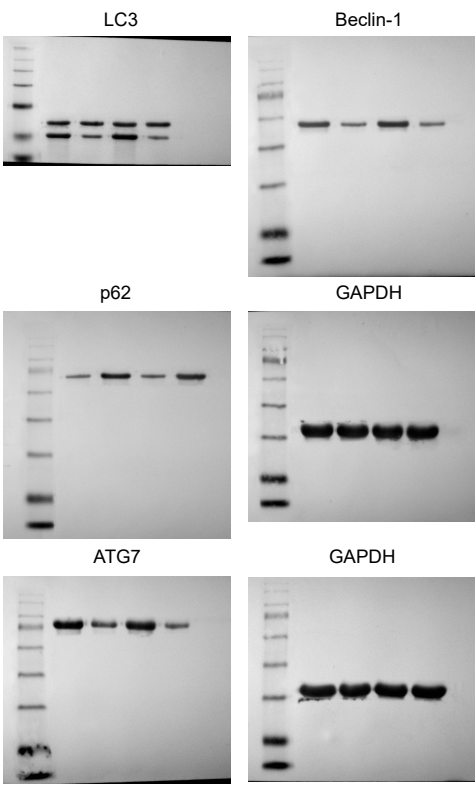

FIG4C

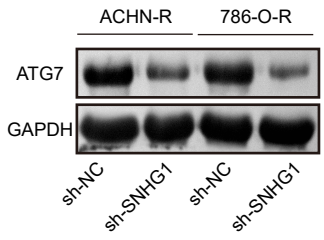

FIG6D

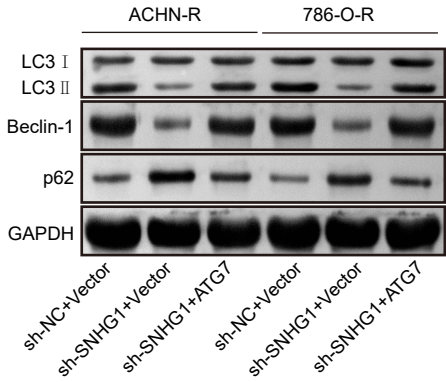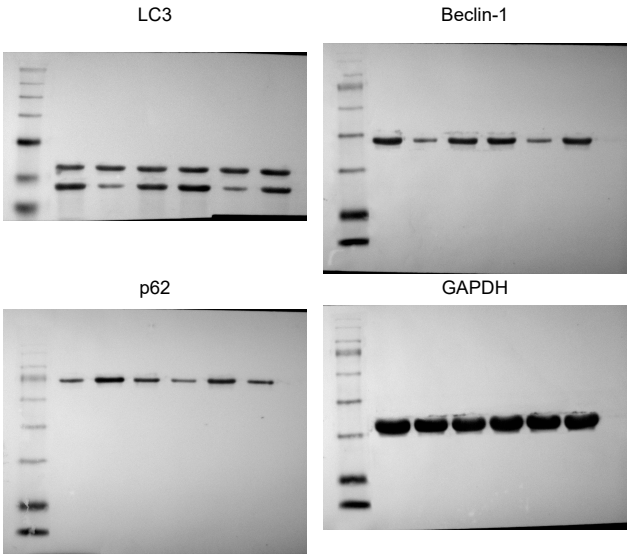

FIG8E

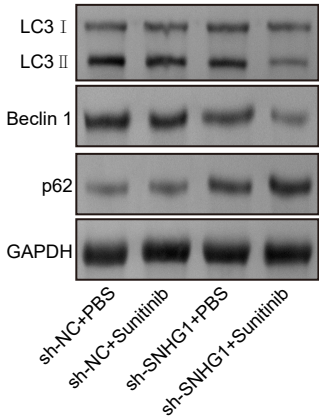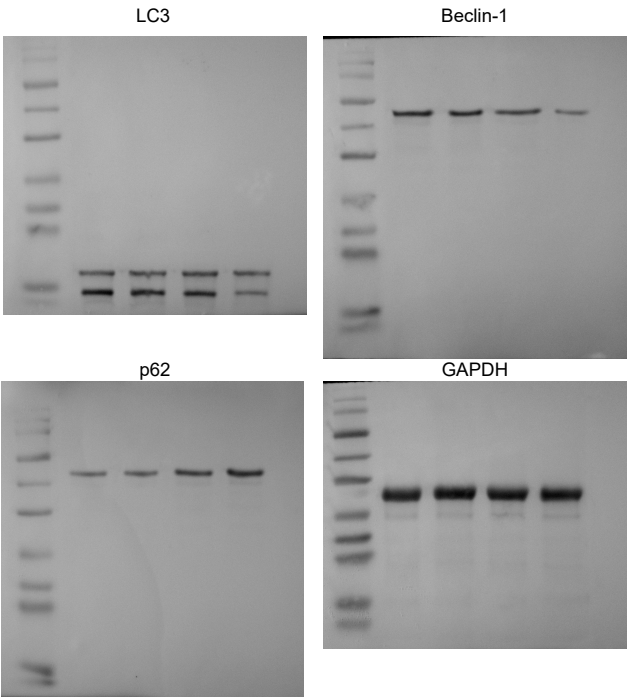

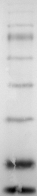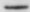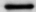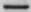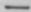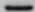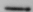

Supplement: Supplementary file 2 — Supplementary materials for western blot [file 41420_2024_2021_MOESM2_ESM.pdf]
